# Accuracy of iodine quantification using dual energy CT in latest generation dual source and dual layer CT

**DOI:** 10.1007/s00330-017-4752-9

**Published:** 2017-02-06

**Authors:** Gert Jan Pelgrim, Robbert W. van Hamersvelt, Martin J. Willemink, Bernhard T. Schmidt, Thomas Flohr, Arnold Schilham, Julien Milles, Matthijs Oudkerk, Tim Leiner, Rozemarijn Vliegenthart

**Affiliations:** 1 Center for Medical Imaging – North East Netherlands, University of Groningen, University Medical Center Groningen, P.O. Box EB44, Hanzeplein 1, 9713 GZ Groningen, The Netherlands; 2 Department of Radiology, Utrecht University Medical Center, Utrecht, The Netherlands; 3 Siemens Healthcare GmbH, Forchheim, Germany; 4 Philips Healthcare, Best, The Netherlands; 5 Department of Radiology, University of Groningen, University Medical Center Groningen, Groningen, The Netherlands

**Keywords:** Tomography, x-ray computed, Absorptiometry, photon, Myocardial perfusion imaging, Iodine, Phantoms, imaging

## Abstract

**Objective:**

To determine the accuracy of iodine quantification with dual energy computed tomography (DECT) in two high-end CT systems with different spectral imaging techniques.

**Methods:**

Five tubes with different iodine concentrations (0, 5, 10, 15, 20 mg/ml) were analysed in an anthropomorphic thoracic phantom. Adding two phantom rings simulated increased patient size. For third-generation dual source CT (DSCT), tube voltage combinations of 150Sn and 70, 80, 90, 100 kVp were analysed. For dual layer CT (DLCT), 120 and 140 kVp were used. Scans were repeated three times. Median normalized values and interquartile ranges (IQRs) were calculated for all kVp settings and phantom sizes.

**Results:**

Correlation between measured and known iodine concentrations was excellent for both systems (*R* = 0.999–1.000, *p* < 0.0001). For DSCT, median measurement errors ranged from −0.5% (IQR −2.0, 2.0%) at 150Sn/70 kVp and −2.3% (IQR −4.0, −0.1%) at 150Sn/80 kVp to −4.0% (IQR −6.0, −2.8%) at 150Sn/90 kVp. For DLCT, median measurement errors ranged from −3.3% (IQR −4.9, −1.5%) at 140 kVp to −4.6% (IQR −6.0, −3.6%) at 120 kVp. Larger phantom sizes increased variability of iodine measurements (*p* < 0.05).

**Conclusion:**

Iodine concentration can be accurately quantified with state-of-the-art DECT systems from two vendors. The lowest absolute errors were found for DSCT using the 150Sn/70 kVp or 150Sn/80 kVp combinations, which was slightly more accurate than 140 kVp in DLCT.

***Key Points*:**

• *High*-*end CT scanners allow accurate iodine quantification using different DECT techniques*.

• *Lowest measurement error was found in scans with largest photon energy separation*.

• *Dual*-*source CT quantified iodine slightly more accurately than dual layer CT*.

## Introduction

Coronary artery disease (CAD) remains a widespread disease in the Western society. Moreover, the impact of CAD will increase in the next decades because of the epidemic of obesity. Cardiovascular disease (CVD) is the primary cause of death in the USA and overall annual economic burden is substantial [1]. In 2010, medical costs with a direct relation to cardiovascular disease were US$273 billion for the USA only [2].

Computed tomography (CT) angiography is a proven technique for the detection of coronary stenosis with high sensitivity and negative predictive value [3]. However, especially in cases with intermediate stenosis it is often difficult to determine whether a stenosis is significant or not. Myocardial perfusion scanning under stress can help in the diagnostic process of determining the significance of any indeterminate stenosis, e.g. by single-photon emission computed tomography (SPECT), positron emission tomography (PET) or magnetic resonance imaging (MRI) [4]. Recent developments have renewed interest for perfusion analysis using CT. One of the methods to evaluate myocardial ischemia with CT is dual energy CT (DECT). In DECT, high energy x-ray photons are differentiated from low energy x-ray photons. Because attenuation of materials and tissues differs at different photon energies [5–7], DECT allows for quantification of materials and tissues. By quantifying iodinated contrast media the DECT technique does not provide direct information about the blood flow but provides an estimate of contrast distribution across the myocardium at one point in time. However, iodine distribution has a direct relation to the myocardial blood flow, and thus provides a semi-quantitative biomarker for myocardial perfusion

Currently several DECT methods are available of which two are analysed in this study: the dual source CT (DSCT) whereby two x-ray tubes are operated at separate tube voltages and the dual layer spectral CT (DLCT) where low energy photons are absorbed in the first detector layer and high energy photons in the second detector layer [5, 8, 9]. Koonce et al. found good stability and accuracy of the iodine content determination on DSCT scanners, using options available at that time [10]. Since then, third-generation DSCT has become available, providing the possibility to use a tin filter with a high tube voltage of 150 kVp, resulting in a narrow spectrum of photons at a higher energy and larger spectral separation with the low tube voltages (down to 70 kVp). Furthermore, the first generation of DLCT has been recently introduced in the clinic. It is expected that a high tube voltage (140 kVp) results in better spectral separation compared to a lower tube voltage (120 kVp) and thus in more accurate iodine quantification; however, the performance in iodine quantification using DLCT is still unknown. Therefore the aim of the current study was to determine the accuracy of the quantification of iodine concentrations on third-generation DSCT and first-generation DLCT in a phantom.

## Materials and methods

### Phantom description

**Fig. 1:**
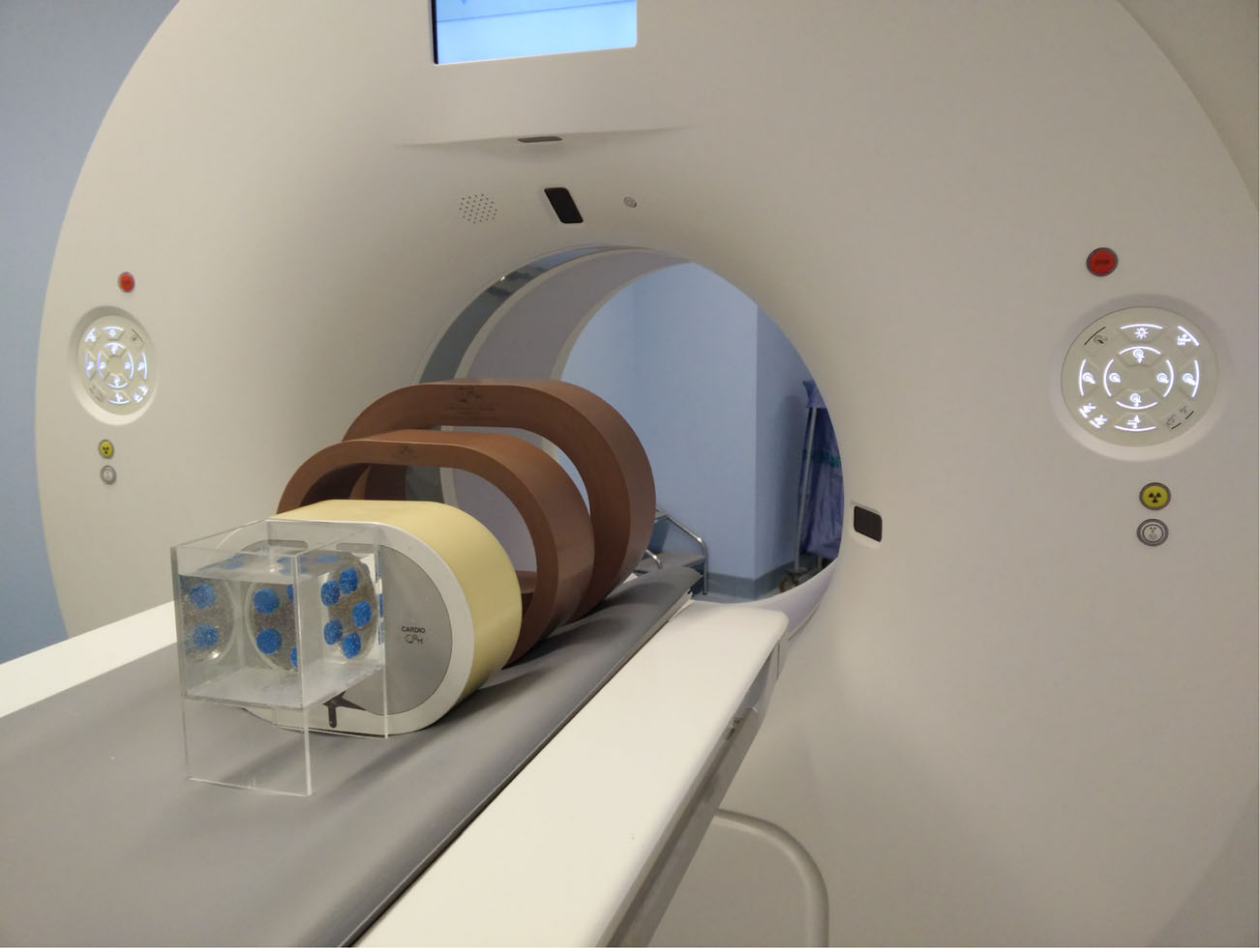
QRM iodine quantification setup, including the fat rings and the iodine tubes surrounded by water

An anthropomorphic phantom (Cardio CT phantom, QRM, Möhrendorf, Germany) was used to simulate the human thorax. This thorax phantom contained artificial lungs, spine, body fat and a cavity at the position of the heart. The cavity was filled with a Perspex holder carrying five separate tubes. These 15-ml tubes were filled with diluted contrast solutions (Hexabrix 320 mg iodine/ml, Guerbet, Paris, France), resulting in concentrations of 0, 5, 10, 15 and 20 mg of iodine per ml, one tube for each concentration. The Perspex holder was placed in a water container in the centre of the cardiac cavity, surrounding the iodine tubes and the Perspex holder with water. Different patient sizes were simulated using extension rings with densities comparable to fat (Extension rings, QRM, Möhrendorf, Germany), which were placed around the thorax. Two separate fat rings were used, providing image data of simulated small, medium and large patient sizes (Fig. 1).

### Image acquisition

Image acquisition was performed in dual energy mode on third-generation DSCT (SOMATOM Force, Siemens Healthcare, Forchheim, Germany) and first-generation DLCT (IQon spectral CT, Philips Healthcare, Best, the Netherlands). For DSCT, 150 kVp with tin (Sn) filter was used as the high tube voltage in combination with low tube voltages of 70, 80, 90 and 100 kVp in spiral scan mode. For DLCT, tube voltages of 140 and 120 kVp were used in spiral scan mode. Tube currents for DSCT were 165 mAs/rot at low tube voltage and 150 mAs/rot at the high tube voltage (vendor-recommended protocol). Tube current of 200 mAs was used for DLCT, as part of a clinical use protocol. This mAs was selected in order to acquire DECT data at comparable radiation doses for DSCT and DLCT. For DSCT, mean CT dose index (CTDI_vol_) gradually increased when raising the low tube voltages of the DSCT combinations, from 18.7 mGy for 150Sn/70 kVp and 22.1 mGy for 150Sn/80 kVp to 26.9 mGy for 150Sn/90 kVp and 32.2 mGy for 150Sn/100 kVp. For DLCT, mean CTDI_vol_ for the 140 kVp tube voltage was 26.0 mGy, while 120-kVp tube voltage resulted in a CTDI_vol_ of 18.1 mGy. All acquisitions were repeated three times with small translations and rotations between the separate scan repetitions. Various grades of iterative reconstruction (IR) were used to analyse the influence of increased IR on iodine quantification. Third-generation DSCT data were reconstructed with a previously described IR algorithm (ADMIRE, Siemens Healthcare, Forchheim, Germany) [11, 12], whereas DLCT data were reconstructed with a model-based iterative reconstruction algorithm taking into account anti-correlated noise. DSCT data were reconstructed using four settings: IR grades 0, 1, 3 and 5. DLCT data were reconstructed using spectral levels of 0, 2, 4 and 6. For DSCT, scans were acquired with a gantry rotation time of 250 ms, a pitch of 0.19 and a detector collimation of 2 × 64 × 0.6 mm. DLCT scans were acquired with a gantry rotation time of 330 ms, a pitch of 0.18 and a detector collimation of 64 × 0.625 mm.

### Image analysis and iodine quantification

Images were reconstructed at 3.0 mm slice thickness with an increment of 1.5 mm using the standard kernel settings. The images of the separate systems were analysed using vendor-specific software, Syngo.via software VB10 (Siemens, Forchheim, Germany) for DSCT and Spectral Diagnostic Suite (Philips Healthcare, Best, the Netherlands) for DLCT. In Syngo.via a dedicated DECT pack ‘Special’ was used and analysis was performed in ‘virtual unenhanced’ setting. In the Spectral Diagnostic Suite, image analysis is performed natively in spectral CT mode. For each iodine tube at each scan, one region of interest (ROI) was drawn in the coronal plane to maximize ROI areas; each ROI was at least 5.0 cm^2^. The mean iodine concentration in the ROI was calculated by the dedicated software packages of each vendor. DECT iodine concentration measurements were compared to the known iodine contrast concentration. Normalized difference of concentration measurements was calculated by dividing the difference between the CT-measured and the known concentration by the known concentration for all separate measurements.

### Statistical analysis

Statistical analysis was performed using SPSS version 23 (IBM Corp, Armonk, NY, USA). Pearson correlation between the CT-measured and known iodine concentration was determined for all DECT scans. Median absolute measurement errors, as well as normalized measurement errors, were calculated for each possibly influencing factor, e.g. number of fat rings, IR grade and tube voltage combination. Kendall’s Tau b (τ_b_) as a measure of trends and correlation was determined between IR grades and iodine measures on both CT systems, and between patient size and iodine measures on the two CT systems. Median normalized iodine measurement errors were used to identify the CT protocols with the smallest iodine measurement error and interquartile ranges (IQR) compared to known concentrations. Furthermore, normalized differences in iodine quantification were compared for scan protocols with lowest measurement error at both CT systems using the Mann–Whitney *U* test. Normalized differences in iodine quantification were compared for different patient sizes using the Kruskal–Wallis test. Variances were compared for several kVp combinations and patient sizes in order to acquire information about the stability and consistency of the separate DECT protocols. Variances in normalized iodine measurement differences were compared for the scan protocols with lowest measurement error using Levene’s test for equality of variances.

## Results

### Third-generation DSCT

**Fig. 2:**
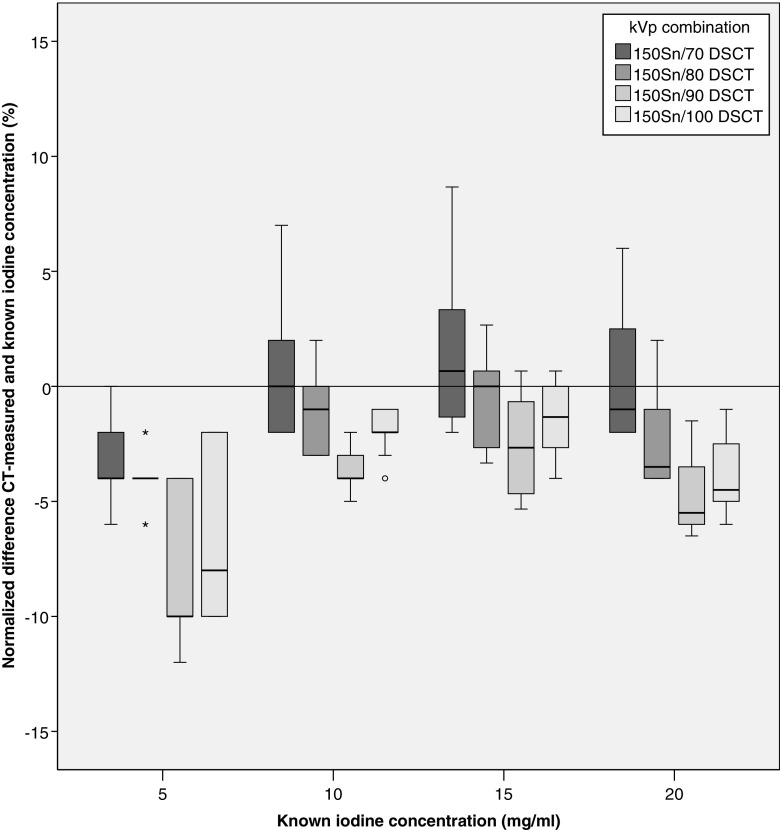
Normalized difference between CT-measured and known iodine concentrations is shown for each DSCT kVp combination by true iodine concentration

In total, 720 measurements were performed on DSCT scan data (3 repetitions × 5 concentration grades × 3 patient sizes × 4 iterative reconstruction grades × 4 kVp combinations). Correlation between measured and true iodine concentration was excellent at all kVp combinations (*R* = 0.999–1.000, *p* < 0.0001). For the 150Sn/70 kVp combination, the measurement error was smallest with a median difference of −0.5% (IQR −2.0, 2.0%) and a median absolute error of −0.1 mg/ml (IQR −0.2, 0.2 mg/ml). The median difference was slightly larger for 150Sn/80 kVp and 150Sn/100 kVp, −2.3% (IQR −4.0%, −0.1%) and −2.3% (IQR −5.0, −1.1) with an absolute error of −0.2 mg/ml (IQR −0.3, −0.1 mg/ml) and −0.2 mg/ml (IQR −0.5, −0.1 mg/ml). The other kVp combinations showed more underestimation (Fig. 2, Table 1).

**Table 1:** Median CT-measured iodine concentration and normalized differences between CT-measured and known iodine concentration are shown for both DSCT and DLCT

	Dual source CT				Dual layer CT	
Iodine concentration	150Sn/70	150Sn/80	150Sn/90	150Sn/100	120 kVp	140 kVp
0 mg/ml (%)	−0.1	−0.1	−0.1	−0.1	0	0
5 mg/ml (%)	4.8 (−4%)	4.8 (−4%)	4.5 (−10%)	4.6 (−8%)	4.5 (−10%)	4.7 (−6%)
10 mg/ml (%)	10.0 (0%)	9.9 (−1%)	9.6 (−4%)	9.8 (−2%)	9.6 (−4%)	9.7 (−3%)
15 mg/ml (%)	15.1 (1%)	15.0 (0%)	14.6 (−3%)	14.8 (−1%)	14.3 (−5%)	14.5 (−3%)
20 mg/ml (%)	19.8 (−1%)	19.3 (−4%)	18.9 (−6%)	19.1 (−5%)	19.3 (−4%)	19.7 (−2%)
Median diff concentration grades mg/ml (%)	−0.1 (−1%)	−0.2 (−2%)	−0.4 (−4%)	−0.2 (−2%)	−0.5 (−5%)	−0.3 (−3%)

Median difference for the separate kVp combinations is shown

**Fig. 3:**
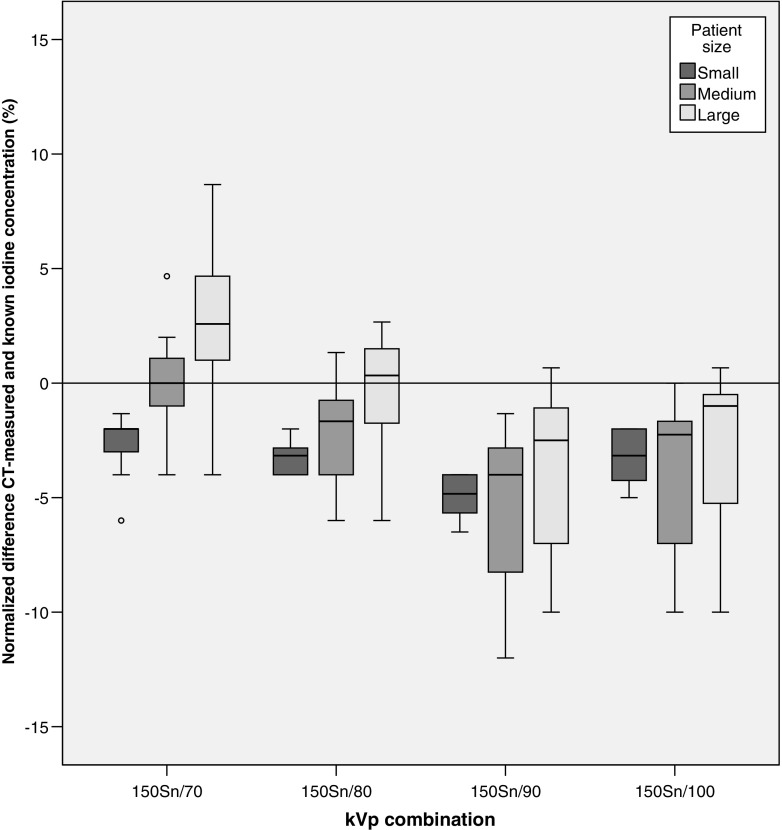
Normalized difference between the CT-measured and known iodine concentration is shown for the kVp combinations available at DSCT by patient size. An increase in CT-determined iodine can be distinguished for all kVp combinations, although the trend was not significant

No significant correlation was found between normalized iodine measurement error and different grades of IR, which implies that the addition of IR does not influence iodine concentration measurements for third-generation DSCT (Kendall τ_b_ test, 150Sn/70 *p* = 0.81, 150Sn/80 *p* = 0.85, 150Sn/90 *p* = 0.66, 150Sn/100 *p* = 0.59). For all kVp combinations, an increase in patient size resulted in slightly higher median iodine measurements; however, there were no significant trends (Kendall τ_b_ test, 150Sn/70 τ_b_ > 0.07, *p* = 0.53; 150Sn/80 τ_b_ > 0.06, *p* = 0.62; 150Sn/90 τ_b_ > 0.05, *p* = 0.69; 150Sn/100 τ_b_ > 0.03, *p* = 0.79) (Fig. 3). For the 150Sn/70, 150Sn/80 and 150Sn/90 kVp combinations, normalized measurement error between CT-determined and known iodine concentration was significantly different between three patient sizes (Kruskal–Wallis test, *p* < 0.05). Only the 150Sn/100 kVp combination showed no significant difference when comparing the normalized measurement error between the three patient sizes (Kruskal–Wallis test, *p* = 0.16). The Levene’s variance test showed no significant difference in variance for normalized measurement error between 150Sn/70 and 150Sn/80 kVp combination in any patient size (small: *p* = 0.06; medium *p* = 0.52; large *p* = 0.46). The 150Sn/100 kVp combination showed significantly larger variances in medium patient size compared to the 150Sn/70 kVp protocol (Levene’s test, *p* < 0.05). Furthermore, the variance of the normalized measurement error of the 150Sn/100 kVp combination was significantly larger in small and medium patient sizes compared to the corresponding patient size groups for the 150Sn/80 kVp protocol (Levene’s test, *p* < 0.05).

### First-generation DLCT

**Fig. 4:**
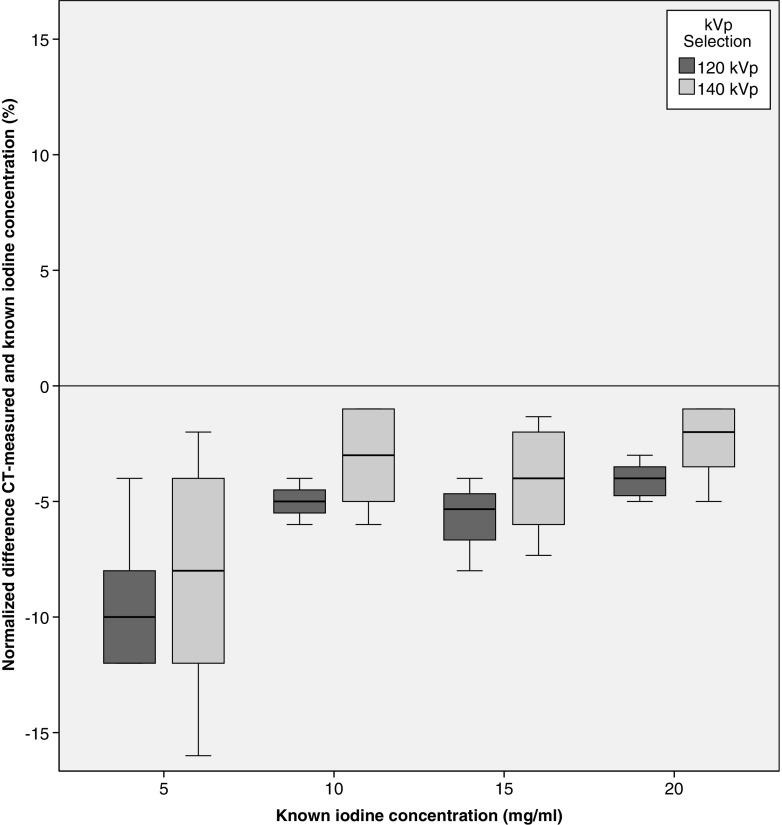
Normalized differences between the CT-measured and known concentration are shown for the DLCT tube voltages by patient size

In total, 360 measurements were performed on DLCT scan data (3 repetitions × 5 concentration grades × 3 patient sizes × 4 iterative reconstruction grades × 2 kVp tube voltages). Correlations between measured and known iodine concentrations were excellent for both kVp combinations (*R* = 0.999–1.000, *p* < 0.0001). Median measurement error for 140 kVp was −4.0% (IQR −6.0, −1.6%) with an absolute median deviation of −0.3 mg/ml (IQR −0.6, −0.1 mg/ml) (Fig. 4, Table 1). The 120 kVp setting showed more underestimation of the iodine content in the tubes (median measurement error, −5.0% (IQR −7.0, −4.0%) median absolute difference, −0.6 mg/ml (IQR −0.8, −0.3 mg/ml) (*p* < 0.05)).

**Fig. 5:**
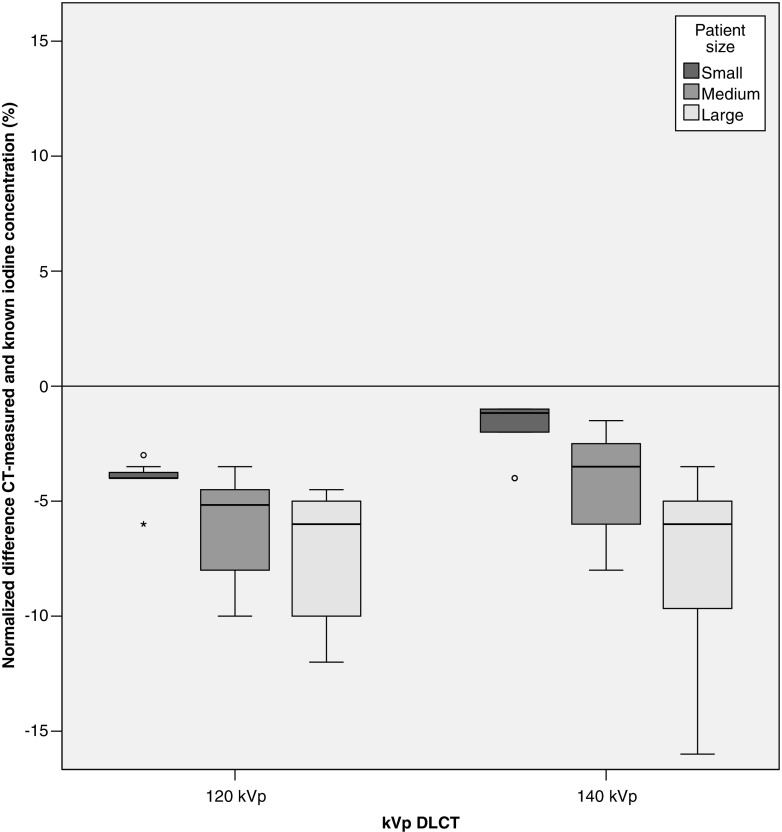
Normalized difference between the CT-measured and known iodine concentration is shown for the kVp combinations available at DLCT by patient size. A decrease in CT-determined iodine can be distinguished for all kVp combinations, although the trend was not significant

Different grades of model-based iterative reconstruction settings did not influence iodine quantification (Kendall τ_b_ test, 120 kVp *p* = 0.80, 140 kVp *p* = 0.59). For both kVp combinations, an increase in patient size resulted in lower measured iodine concentrations, although not significant using Kendall’s τ_b_ test (120 kVp τ_b_ < −0.11, *p* = 0.36; 140 kVp τ_b_ < −0.13 *p* = 0.30) (Fig. 5). Large patient size showed more underestimation of iodine concentrations compared to medium and small patient size for both the tube voltages (Kruskal–Wallis test, *p* < 0.05). No significant difference in variance was found between the two tube voltages in any patient size (Levene’s test, small *p* = 0.25, medium *p* = 0.92, large *p* = 0.50).

### Comparison between third-generation DSCT and first-generation DLCT

A significant difference was found for the normalized difference between CT-measured and known iodine concentration between the DSCT 150Sn/70 and the DLCT 140 kVp tube voltage (Mann–Whitney *U* test, *p* < 0.05), indicating closer agreement with known iodine concentrations for the DSCT 150Sn/70 kVp combination. Similarly, measurement error was smaller for 150Sn/80 kVp DSCT compared to 140 kVp DLCT (Mann–Whitney *U* test, *p* < 0.05). However, no significant difference in normalized measurement error was found when 150Sn/100 kVp DSCT was compared to 140 kVp DLCT (Mann–Whitney *U* test, *p* = 0.21). No significant difference in variances was found for all of the DSCT kVp combinations when compared to DLCT 140 kVp (Levene’s variance test, 150Sn70 vs. 140 kVp: *p* = 0.91; 150Sn80 vs. 140 kVp: *p* = 0.08). Also when analysing patient sizes separately, no significant differences in variance were found (Levene’s variance test, 150Sn70 vs. 140 kVp: small *p* = 0.52, medium *p* = 0.44, large = 0.77; 150Sn80 vs. 140 kVp: small *p* = 0.18, medium *p* = 0.82, large = 0.24).

## Discussion

In this study the accuracy of iodine quantification using DECT was studied in third-generation DSCT system and first-generation DLCT. Using a phantom model, we showed that the correlation of iodine measurements with true concentrations was excellent for both DSCT and DLCT. The results suggest that iodine quantification in the myocardium could be a semi-quantitative surrogate for the quantification of myocardial perfusion, with iodine concentration in the myocardium as an expression of the blood distribution at one moment in time. The most accurate results were found for protocols with the largest photon energy separation, namely 150Sn/70 and 150Sn/80 kVp for DSCT and 140 kVp for DLCT.

Koonce et al. analysed iodine quantification using DECT for first- and second-generation DSCT systems [10]. That study demonstrated stable and accurate results for multiple acquisition protocols. Our results complement that study, with even closer or comparable agreement between CT-measured and known iodine concentration for two new state-of-the-art CT systems, a third-generation DSCT system and a first-generation DLCT system. Another study analysed iodine quantification on single-source CT with a split filter, with measured differences ranging between 2 and 21% for iodine concentrations ranging from 1.2 to 23.5 mg/ml, with absolute differences ranging from 0.1 to 1.6 mg/ml [13]. When comparing these results to our study, one can state that increased spectral separation increases the accuracy of iodine quantification.

Only one study analysed the quantification of iodine in patients using a second-generation DSCT system [14]. The authors concluded that quantification of iodine could be useful for differentiation between normal, ischemic and necrotic myocardium. Reference iodine values for normal and ischemic myocardium were reported to be 2.6 and 2.0 mg/ml, respectively. In our study, the low iodine concentrations in the range of 0 to 5 mg/ml resulted in 0.2 to 0.3 mg/ml underestimations using the optimal kVp combination of 150Sn/70, 150Sn/80 for DSCT and 140 kVp for DLCT. Taking into account the small differences between normal and ischemic myocardium as reported by Delgado et al. and the results provided in this study, one should be careful to crosslink iodine quantification results between scanners and kVp combinations. Results of iodine quantification studies can only be compared when the same scanner type and kVp combination is used.

DECT cannot only be used in quantification of iodine concentration in the myocardium but also has the potential to assess patency of coronary arteries, a standard CT angiography [15, 16]. The use of only a slightly changed cardiac CT protocol and the ability to compare stress images to rest (coronary CTA) images are advantages of DECT compared to dynamic first-pass perfusion CT. Furthermore, radiation dose of a single-shot DECT scan is lower than the radiation dose in dynamic perfusion CT. In comparison to DECT, a major advantage of the dynamic first-pass perfusion CT is the ability to quantify absolute myocardial perfusion, and derive myocardial blood flow and blood volume. However, DECT allows for assessment of a semi-quantitative biomarker of myocardial perfusion, namely myocardial iodine content. Quantification of myocardial iodine content may help in detection of myocardial ischemia [14]. For this purpose, accurate quantification of iodine content, as we have studied, is an important issue. The quantification of iodine using DECT only calculates a semi-quantitative parameter of myocardial perfusion, namely myocardial iodine distribution in milligrams per millilitre. Still, this may be more sensitive to detect myocardial ischemia than mere visual evaluation. In this study, we showed that the ability to quantify iodine is highly accurate. Future studies should show whether quantification of myocardial iodine distribution has additional diagnostic value in patients suspected of myocardial ischemia, beyond visual analysis of myocardial iodine distribution.

It is important to strive for a quantitative analysis of myocardial blood supply. Quantitative analysis may allow more sensitive evaluation of myocardial perfusion deficits instead of relative, visual analysis. This is of particular importance in three-vessel disease, in which the entire myocardium may have reduced perfusion. Three-vessel disease can be missed when using qualitative assessment of relative contrast distribution. Also, quantification of myocardial perfusion parameters, or iodine content, may possibly enable detection of subclinical CAD, in which blood supply is reduced but yet without gross perfusion defects. The added value of DECT using iodine quantification is not only shown for cardiac imaging but several studies have shown added value in oncological imaging, differentiating between renal masses and cysts or assessing treatment response [17–19].

The third-generation DSCT is equipped with a tin filter to harden the high energy (150 kVp) spectrum. This filter absorbs low energy photons before they reach the phantom, causing increased spectral separation. In DSCT, tube voltage, tin filter and tube current can be selected for both tubes separately. Drawbacks of the DSCT technique are the angular offset of the two image datasets, and a smaller field of view for one of the two tubes. Furthermore, the dual energy mode in DSCT needs to be selected, while in DLCT images can always be reconstructed in DECT mode. First-generation DLCT scanning uses a different method to acquire spectral separation. The first layer of detectors registers the low energy photons, while the second layer mainly detects the high energy photons, resulting in spectral separation between the first and second layer of the detector. An increase in tube voltage will result in a larger spectral separation, because more high energy photons will reach the second detector layer. There is no angular offset because the high and low energy datasets are acquired using the same source, and conventional images are created using the information from both high and low energy datasets. Drawbacks of DLCT include the low number of kVp selections (120 and 140 kVp), and the fact that no filter can be selected, as separation takes place at the level of the detector and not at the x-ray tube. This could result in a higher radiation dose. The smaller detector coverage of 64 rows is another drawback of the DLCT technique. Finally, as both the high and low energy datasets are acquired using the same source, spectral separation is lower compared to DSCT and this could result in less accurate iodine quantification, as we have shown in our study. Whether this translates into clinically relevant differences in diagnostic accuracy is unknown.

DECT allows for quantification of iodine concentrations in a static phantom at several patient sizes. However, an increase in patient size will result in differences in quantified iodine for both DSCT and DLCT scanners. Interestingly, increase in patient size tended to have a positive effect (increase in CT-measured iodine) on the normalized differences between CT-determined and known iodine concentration in DSCT, while having a negative effect (decrease in CT-measured iodine) on the quantification of iodine in DLCT. Only the 150Sn/100 tube voltage combination showed no significant difference in normalized difference between CT-determined and known iodine concentration for the three patient sizes. However, this result is likely caused by higher measurement errors in small and medium patient sizes for the 150Sn/100 kVp protocol. Still, one should be careful when comparing the quantification of iodine concentration between different patient sizes for both scan techniques.

The present study has several limitations that need to be recognized. The most important limitation is that measuring iodine in several separate tubes in a thoracic phantom is different from measuring iodine concentrations in patients. Movement of the heart, breathing motion and general movement are not taken into account in this model, but are likely to influence the iodine quantification in patients. These movement problems could be especially problematic when low and high energy data are not acquired at exactly the same time and position, like in most DECT approaches. Water was used for diluting iodine, which is different to clinical practice where surrounding tissue such as calcium and proteins may influence the iodine measurements. Furthermore, the ROIs were larger (over 5.0 cm^2^), which will not always be applicable in clinical practice. Smaller ROIs could increase the measurement error. However, these results form a strong basis from which other studies in iodine quantification using DECT can derive a selection of protocols or kVp combinations. A second limitation is that we limited the dual energy modes to third-generation DSCT and first-generation DLCT, while there are other dual energy methods on the market. The results presented in this study could be different for other methods of iodine quantification using different CT scanners. The third limitation is the range in iodine concentration. The first patient study providing iodine quantification showed that the measured iodine concentration ranges between 1 and 6 mg/ml. This is at the lower end of the concentration range we used in this study. We chose the current range of iodine concentrations to be able to compare to the study by Koonce et al. [10].

## Conclusion

Overall, both third-generation DSCT and first-generation DLCT showed highly accurate quantification of increasing iodine concentrations using DECT protocols. Best results were found for DSCT using the 150Sn/70 or 150Sn/80 kVp combination, which showed lower measurement error than DLCT. For both systems largest energy separation provided the most accurate iodine quantification.

## Abbreviations

CAD: Coronary artery disease
CT: Computed tomography
CVD: Cardiovascular disease
DECT: Dual energy CT
DLCT: Dual layer spectral CT
DSCT: Dual source CT
IQR: Interquartile range
IR: Iterative reconstruction
MRI: Magnetic resonance imaging
PET: Positron emission tomography
ROI: Region of interest
SPECT: Single-photon emission computed tomography
